# (6a*R*,10a*R*)-6,6,9-Trimethyl-3-pentyl-6a,7,8,10a-tetra­hydro-6*H*-benzo[*c*]­chromen-1-yl 4-methyl­benzene­sulfonate

**DOI:** 10.1107/S1600536808022010

**Published:** 2008-08-06

**Authors:** Waseem Gul, Paulo Carvalho, David W. Berberich, Mitchell A. Avery, Mahmoud A. ElSohly

**Affiliations:** a National Center for Natural Products Research, The University of Mississippi, University, MS 38677, USA; b El Sohly Laboratories Inc., 5 Industrial Park Drive, Oxford, MS 38655, USA; c The University of Mississippi, Department of Medicinal Chemistry, 417 Faser Hall, University, MS 38677, USA; d Covidien, 3600 N. 2nd Street, St Louis, MO 63147, USA; e National Center for Natural Products Research, Research Institute of Pharmaceutical Sciences, School of Pharmacy, The University of Mississippi, University, MS 38677, USA; f Department of Chemistry and Biochemistry, The University of Mississippi, University, MS 38677, USA; g National Center for Natural Products Research, Department of Pharmaceutics, School of Pharmacy, The University of Mississippi, University, MS 38677, USA

## Abstract

In the crystal structure of the title compound, C_28_H_36_O_4_S, the *p*-tolyl ring is inclined at 35.8° to the aromatic ring. The cyclohexene ring adopts a boat conformation and the heterocyclic ring is in a slightly distorted screw boat conformation.

## Related literature

For the physiological actions of tetra­hydro­cannabinol (Δ^9^—THC), the most psychologically active constituent of *Cannabis sativa*, see: Mechoulam & Gaoni (1967 ▶). For the synthesis of Δ^9^—THC-tosyl­ate, see: Duchek (2004 ▶).
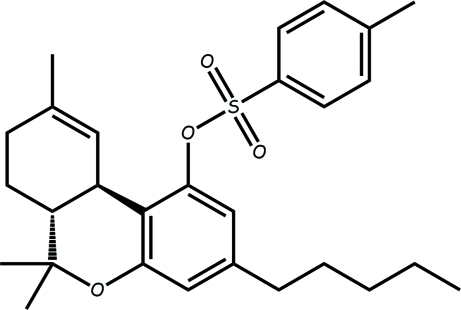


## Experimental

### 

#### Crystal data

C_28_H_36_O_4_S
*M* *_r_* = 468.63
Orthorhombic, 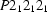

*a* = 9.8759 (2) Å
*b* = 13.2996 (2) Å
*c* = 19.1500 (3) Å
*V* = 2515.27 (7) Å^3^
*Z* = 4
Cu *K*α radiation
μ = 1.39 mm^−1^
*T* = 100 K
0.19 × 0.17 × 0.16 mm

#### Data collection

Bruker SMART CCD area-detector diffractometer
Absorption correction: none
47731 measured reflections
4562 independent reflections
4438 reflections with *I* > 2σ(*I*)
*R*_int_ = 0.034

#### Refinement

*R*[*F* ^2^ > 2σ(*F* ^2^)] = 0.026
*wR*(*F* ^2^) = 0.069
*S* = 1.04
4562 reflections
303 parameters
H-atom parameters constrained
Δρ_max_ = 0.25 e Å^−3^
Δρ_min_ = −0.24 e Å^−3^
Absolute structure: Flack (1983 ▶), 1965 Friedel pairs
Flack parameter: 0.023 (11)

### 

Data collection: *SMART* (Bruker, 2005 ▶); cell refinement: *SAINT* (Bruker, 2003 ▶); data reduction: *SAINT*; program(s) used to solve structure: *SHELXS97* (Sheldrick, 2008 ▶); program(s) used to refine structure: *SHELXL97* (Sheldrick, 2008 ▶); molecular graphics: *SHELXTL* (Sheldrick, 2008 ▶); software used to prepare material for publication: *SHELXTL*.

## Supplementary Material

Crystal structure: contains datablocks I, global. DOI: 10.1107/S1600536808022010/nc2105sup1.cif

Structure factors: contains datablocks I. DOI: 10.1107/S1600536808022010/nc2105Isup2.hkl

Additional supplementary materials:  crystallographic information; 3D view; checkCIF report

## Figures and Tables

**Fig. 1.**
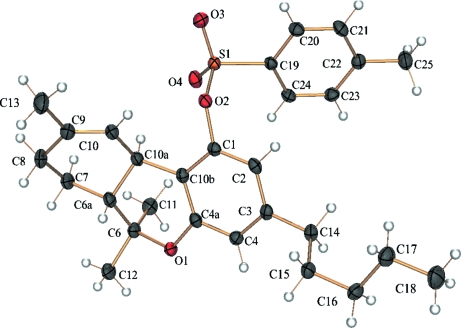
Crystal structure of the title compound with labelling and displacement ellipsoids drawn at the 50% probability level.

## supplementary crystallographic information

### Experimental

Δ^9 ^– Tetrahydrocannabinol tosylate (*p*-tosyl-Δ^9^—THC), was synthesized according to Duchek (2004).

### Refinement

All H atoms were located in difference maps and treated as riding atoms, with the following distance restraints: C—H = 0.93 Å, *U*_iso_=1.2Ueq (C) for C*sp*_2_, C—H = 0.98 Å, *U*_iso_ = 1.2Ueq (C) for CH, C—H = 0.97 Å, *U*_iso_ = 1.2Ueq (C) for CH_2_, C—H = 0.96 Å, *U*_iso_ = 1.5Ueq (C) for CH_3_.

### Crystal data

**Table:**

C_28_H_36_O_4_S	*F*_000_ = 1008
*M**_r_* = 468.63	*D*_x_ = 1.238 Mg m^−^^3^
Orthorhombic, *P*2_1_2_1_2_1_	Cu *K*α radiation λ = 1.54178 Å
Hall symbol: P 2ac 2ab	Cell parameters from 9942 reflections
*a* = 9.8759 (2) Å	θ = 4.1–67.4º
*b* = 13.2996 (2) Å	µ = 1.39 mm^−^^1^
*c* = 19.1500 (3) Å	*T* = 100 K
*V* = 2515.27 (7) Å^3^	Blocks, colourless
*Z* = 4	0.19 × 0.17 × 0.16 mm

### Data collection

**Table:**

Bruker SMART CCD area-detector diffractometer	4438 reflections with *I* > 2σ(*I*)
Monochromator: graphite	*R*_int_ = 0.034
*T* = 100 K	θ_max_ = 68.0º
φ and ω scans	θ_min_ = 4.1º
Absorption correction: none	*h* = −11→11
47731 measured reflections	*k* = −15→15
4562 independent reflections	*l* = −23→23

### Refinement

**Table:**

Refinement on *F*^2^	Hydrogen site location: inferred from neighbouring sites
Least-squares matrix: full	H-atom parameters constrained
*R*[*F*^2^ > 2σ(*F*^2^)] = 0.027	*w* = 1/[σ^2^(*F*_o_^2^) + (0.0429*P*)^2^ + 0.4744*P*] where *P* = (*F*_o_^2^ + 2*F*_c_^2^)/3
*wR*(*F*^2^) = 0.069	(Δ/σ)_max_ = 0.002
*S* = 1.04	Δρ_max_ = 0.25 e Å^−^^3^
4562 reflections	Δρ_min_ = −0.24 e Å^−^^3^
303 parameters	Extinction correction: none
Primary atom site location: structure-invariant direct methods	Absolute structure: Flack (1983), 1965 Friedel pairs
Secondary atom site location: difference Fourier map	Flack parameter: 0.023 (11)

### Special details

**Table:**

Geometry. All e.s.d.'s (except the e.s.d. in the dihedral angle between two l.s. planes)are estimated using the full covariance matrix. The cell e.s.d.'s are takeninto account individually in the estimation of e.s.d.'s in distances, anglesand torsion angles; correlations between e.s.d.'s in cell parameters are onlyused when they are defined by crystal symmetry. An approximate (isotropic)treatment of cell e.s.d.'s is used for estimating e.s.d.'s involving l.s. planes.
Refinement. Refinement of *F*^2^ against ALL reflections. The weighted *R*-factor *wR* andgoodness of fit *S* are based on *F*^2^, conventional *R*-factors *R* are basedon *F*, with *F* set to zero for negative *F*^2^. The threshold expression of*F*^2^ > σ(*F*^2^) is used only for calculating *R*-factors(gt) *etc*. and isnot relevant to the choice of reflections for refinement. *R*-factors basedon *F*^2^ are statistically about twice as large as those based on *F*, and *R*-factors based on ALL data will be even larger.

### Fractional atomic coordinates and isotropic or equivalent isotropic displacement parameters (Å^2^)

**Table:**

	*x*	*y*	*z*	*U*_iso_*/*U*_eq_	
S1	0.46490 (3)	0.05656 (2)	1.076895 (17)	0.01846 (9)	
C1	0.47927 (14)	0.18406 (10)	0.97409 (7)	0.0172 (3)	
C2	0.47639 (14)	0.28371 (11)	0.99381 (7)	0.0187 (3)	
H2	0.5205	0.3046	1.0342	0.022*	
C3	0.40657 (14)	0.35294 (11)	0.95249 (7)	0.0186 (3)	
C4	0.34101 (15)	0.31759 (11)	0.89315 (7)	0.0189 (3)	
H4	0.2911	0.3621	0.8659	0.023*	
C7	0.39042 (17)	−0.07962 (11)	0.78957 (8)	0.0262 (3)	
H7A	0.3350	−0.1182	0.8216	0.031*	
H7B	0.3527	−0.0869	0.7431	0.031*	
C9	0.61971 (16)	−0.07895 (11)	0.84941 (8)	0.0244 (3)	
C10	0.57509 (15)	−0.00757 (11)	0.89264 (7)	0.0207 (3)	
H10	0.6326	0.0152	0.9277	0.025*	
C8	0.53477 (18)	−0.11963 (12)	0.79053 (8)	0.0291 (3)	
H8A	0.5319	−0.1923	0.7941	0.035*	
H8B	0.5780	−0.1029	0.7466	0.035*	
C6	0.25642 (15)	0.08708 (11)	0.79805 (8)	0.0213 (3)	
C4A	0.34857 (14)	0.21680 (11)	0.87368 (7)	0.0184 (3)	
C10A	0.43477 (14)	0.03799 (10)	0.88715 (7)	0.0189 (3)	
H10A	0.3732	−0.0034	0.9152	0.023*	
C10B	0.42230 (14)	0.14608 (10)	0.91271 (7)	0.0178 (3)	
C19	0.40283 (15)	0.14829 (10)	1.13478 (7)	0.0176 (3)	
C20	0.47260 (15)	0.16732 (11)	1.19667 (7)	0.0208 (3)	
H20	0.5532	0.1340	1.2065	0.025*	
C24	0.28354 (15)	0.19860 (11)	1.11914 (7)	0.0199 (3)	
H24	0.2371	0.1851	1.0779	0.024*	
C21	0.42018 (16)	0.23624 (12)	1.24306 (8)	0.0228 (3)	
H21	0.4649	0.2478	1.2850	0.027*	
C14	0.40301 (15)	0.46315 (10)	0.97123 (7)	0.0216 (3)	
H14A	0.4323	0.4713	1.0193	0.026*	
H14B	0.3105	0.4872	0.9679	0.026*	
C11	0.13920 (16)	0.05062 (12)	0.84292 (8)	0.0264 (3)	
H11A	0.1622	0.0579	0.8914	0.040*	
H11B	0.1214	−0.0189	0.8329	0.040*	
H11C	0.0600	0.0898	0.8328	0.040*	
C23	0.23487 (16)	0.26903 (11)	1.16577 (8)	0.0221 (3)	
H23	0.1559	0.3040	1.1551	0.027*	
C22	0.30153 (15)	0.28894 (11)	1.22843 (8)	0.0210 (3)	
C18	0.5664 (2)	0.77077 (13)	1.03217 (10)	0.0409 (5)	
H18A	0.5992	0.8127	0.9951	0.061*	
H18B	0.6205	0.7812	1.0732	0.061*	
H18C	0.4738	0.7876	1.0422	0.061*	
C25	0.24424 (18)	0.36377 (13)	1.27916 (9)	0.0301 (4)	
H25A	0.1675	0.3349	1.3025	0.045*	
H25B	0.2166	0.4231	1.2544	0.045*	
H25C	0.3121	0.3812	1.3130	0.045*	
C15	0.49335 (15)	0.52705 (10)	0.92386 (8)	0.0227 (3)	
H15A	0.4620	0.5208	0.8761	0.027*	
H15B	0.5852	0.5013	0.9258	0.027*	
C17	0.57512 (18)	0.66107 (12)	1.01015 (9)	0.0293 (4)	
H17A	0.5419	0.6191	1.0479	0.035*	
H17B	0.6693	0.6438	1.0022	0.035*	
C16	0.49424 (17)	0.63855 (11)	0.94437 (8)	0.0260 (3)	
H16A	0.5317	0.6773	0.9060	0.031*	
H16B	0.4016	0.6606	0.9513	0.031*	
C12	0.21926 (17)	0.08792 (12)	0.72084 (8)	0.0273 (3)	
H12A	0.1454	0.1336	0.7133	0.041*	
H12B	0.1928	0.0215	0.7067	0.041*	
H12C	0.2961	0.1091	0.6939	0.041*	
O2	0.55008 (10)	0.11806 (7)	1.01992 (5)	0.0185 (2)	
O3	0.56579 (11)	−0.00225 (8)	1.11089 (5)	0.0271 (2)	
O4	0.35208 (11)	0.00985 (8)	1.04341 (5)	0.0240 (2)	
C13	0.75923 (18)	−0.12306 (14)	0.85622 (9)	0.0344 (4)	
H13A	0.8061	−0.0908	0.8940	0.052*	
H13B	0.8084	−0.1127	0.8136	0.052*	
H13C	0.7523	−0.1938	0.8654	0.052*	
C6A	0.38882 (15)	0.03099 (10)	0.81073 (7)	0.0201 (3)	
H6A	0.4582	0.0647	0.7827	0.024*	
O1	0.28074 (11)	0.19339 (8)	0.81346 (5)	0.0207 (2)	

### Atomic displacement parameters (Å^2^)

**Table:**

	*U*^11^	*U*^22^	*U*^33^	*U*^12^	*U*^13^	*U*^23^
S1	0.02166 (17)	0.01704 (15)	0.01669 (15)	0.00077 (14)	0.00114 (13)	0.00015 (13)
C1	0.0154 (7)	0.0200 (7)	0.0162 (6)	−0.0006 (6)	0.0033 (5)	0.0013 (5)
C2	0.0174 (7)	0.0236 (7)	0.0149 (6)	−0.0041 (6)	0.0019 (5)	−0.0024 (5)
C3	0.0178 (7)	0.0188 (7)	0.0191 (7)	−0.0019 (6)	0.0064 (5)	−0.0007 (5)
C4	0.0189 (7)	0.0188 (7)	0.0190 (7)	−0.0008 (6)	0.0023 (5)	0.0015 (6)
C7	0.0351 (9)	0.0208 (8)	0.0227 (7)	−0.0001 (7)	−0.0047 (6)	−0.0053 (6)
C9	0.0272 (8)	0.0229 (8)	0.0231 (7)	0.0022 (6)	0.0012 (6)	−0.0001 (6)
C10	0.0240 (7)	0.0203 (7)	0.0177 (6)	0.0003 (6)	−0.0006 (5)	−0.0013 (6)
C8	0.0382 (9)	0.0228 (7)	0.0263 (8)	0.0047 (7)	0.0008 (7)	−0.0079 (6)
C6	0.0252 (8)	0.0166 (7)	0.0221 (7)	−0.0025 (6)	−0.0026 (6)	−0.0028 (5)
C4A	0.0174 (7)	0.0215 (7)	0.0162 (7)	−0.0024 (6)	0.0029 (5)	−0.0014 (5)
C10A	0.0221 (7)	0.0184 (7)	0.0161 (6)	−0.0019 (6)	0.0006 (5)	−0.0014 (5)
C10B	0.0172 (7)	0.0182 (7)	0.0179 (7)	−0.0019 (5)	0.0027 (5)	−0.0010 (5)
C19	0.0195 (7)	0.0185 (7)	0.0148 (6)	−0.0010 (6)	0.0024 (5)	0.0011 (5)
C20	0.0174 (7)	0.0258 (7)	0.0193 (7)	0.0010 (6)	−0.0017 (6)	0.0014 (5)
C24	0.0197 (7)	0.0230 (7)	0.0169 (7)	−0.0010 (6)	−0.0026 (6)	0.0006 (5)
C21	0.0223 (8)	0.0286 (8)	0.0175 (7)	−0.0048 (6)	−0.0032 (6)	−0.0024 (6)
C14	0.0220 (7)	0.0203 (7)	0.0225 (7)	−0.0008 (6)	0.0022 (6)	−0.0038 (5)
C11	0.0233 (8)	0.0255 (7)	0.0303 (8)	−0.0017 (7)	−0.0014 (6)	−0.0008 (6)
C23	0.0197 (7)	0.0242 (7)	0.0225 (7)	0.0039 (6)	−0.0001 (6)	0.0031 (6)
C22	0.0224 (8)	0.0187 (7)	0.0220 (7)	−0.0032 (6)	0.0024 (6)	0.0006 (6)
C18	0.0586 (13)	0.0257 (8)	0.0384 (10)	−0.0098 (8)	0.0003 (9)	−0.0062 (7)
C25	0.0326 (9)	0.0304 (8)	0.0273 (8)	0.0026 (7)	−0.0007 (7)	−0.0070 (7)
C15	0.0264 (7)	0.0193 (7)	0.0224 (7)	−0.0013 (6)	0.0000 (6)	−0.0009 (6)
C17	0.0332 (9)	0.0217 (8)	0.0329 (9)	−0.0040 (7)	−0.0023 (7)	−0.0014 (6)
C16	0.0321 (9)	0.0176 (7)	0.0284 (8)	−0.0006 (6)	−0.0016 (6)	0.0021 (6)
C12	0.0336 (9)	0.0248 (7)	0.0235 (8)	0.0005 (7)	−0.0070 (6)	−0.0050 (6)
O2	0.0180 (5)	0.0210 (5)	0.0164 (5)	0.0007 (4)	0.0003 (4)	−0.0004 (4)
O3	0.0329 (6)	0.0244 (5)	0.0242 (5)	0.0065 (5)	0.0008 (4)	0.0025 (4)
O4	0.0277 (6)	0.0222 (5)	0.0220 (5)	−0.0061 (4)	0.0039 (4)	−0.0029 (4)
C13	0.0325 (9)	0.0362 (9)	0.0346 (9)	0.0108 (7)	0.0016 (7)	−0.0088 (7)
C6A	0.0232 (8)	0.0195 (7)	0.0175 (7)	−0.0032 (6)	0.0007 (6)	−0.0031 (5)
O1	0.0271 (5)	0.0172 (5)	0.0179 (5)	−0.0009 (4)	−0.0047 (4)	−0.0017 (4)

### Geometric parameters (Å, °)

**Table:**

S1—O3	1.4243 (11)	C20—H20	0.9300
S1—O4	1.4277 (11)	C24—C23	1.380 (2)
S1—O2	1.6022 (10)	C24—H24	0.9300
S1—C19	1.7586 (14)	C21—C22	1.394 (2)
C1—C2	1.3783 (19)	C21—H21	0.9300
C1—C10B	1.3976 (19)	C14—C15	1.5301 (19)
C1—O2	1.4247 (17)	C14—H14A	0.9700
C2—C3	1.396 (2)	C14—H14B	0.9700
C2—H2	0.9300	C11—H11A	0.9600
C3—C4	1.390 (2)	C11—H11B	0.9600
C3—C14	1.5095 (19)	C11—H11C	0.9600
C4—C4A	1.393 (2)	C23—C22	1.394 (2)
C4—H4	0.9300	C23—H23	0.9300
C7—C8	1.522 (2)	C22—C25	1.501 (2)
C7—C6A	1.5260 (19)	C18—C17	1.521 (2)
C7—H7A	0.9700	C18—H18A	0.9600
C7—H7B	0.9700	C18—H18B	0.9600
C9—C10	1.335 (2)	C18—H18C	0.9600
C9—C13	1.503 (2)	C25—H25A	0.9600
C9—C8	1.506 (2)	C25—H25B	0.9600
C10—C10A	1.516 (2)	C25—H25C	0.9600
C10—H10	0.9300	C15—C16	1.5341 (19)
C8—H8A	0.9700	C15—H15A	0.9700
C8—H8B	0.9700	C15—H15B	0.9700
C6—O1	1.4643 (17)	C17—C16	1.521 (2)
C6—C11	1.521 (2)	C17—H17A	0.9700
C6—C12	1.523 (2)	C17—H17B	0.9700
C6—C6A	1.525 (2)	C16—H16A	0.9700
C4A—O1	1.3694 (17)	C16—H16B	0.9700
C4A—C10B	1.405 (2)	C12—H12A	0.9600
C10A—C10B	1.5236 (18)	C12—H12B	0.9600
C10A—C6A	1.5350 (18)	C12—H12C	0.9600
C10A—H10A	0.9800	C13—H13A	0.9600
C19—C24	1.388 (2)	C13—H13B	0.9600
C19—C20	1.394 (2)	C13—H13C	0.9600
C20—C21	1.377 (2)	C6A—H6A	0.9800
			
O3—S1—O4	120.81 (7)	C3—C14—H14A	109.1
O3—S1—O2	102.96 (6)	C15—C14—H14A	109.1
O4—S1—O2	109.03 (6)	C3—C14—H14B	109.1
O3—S1—C19	109.68 (7)	C15—C14—H14B	109.1
O4—S1—C19	108.24 (7)	H14A—C14—H14B	107.8
O2—S1—C19	104.96 (6)	C6—C11—H11A	109.5
C2—C1—C10B	124.73 (13)	C6—C11—H11B	109.5
C2—C1—O2	115.71 (12)	H11A—C11—H11B	109.5
C10B—C1—O2	119.53 (12)	C6—C11—H11C	109.5
C1—C2—C3	119.27 (13)	H11A—C11—H11C	109.5
C1—C2—H2	120.4	H11B—C11—H11C	109.5
C3—C2—H2	120.4	C24—C23—C22	121.41 (14)
C4—C3—C2	118.06 (13)	C24—C23—H23	119.3
C4—C3—C14	120.79 (13)	C22—C23—H23	119.3
C2—C3—C14	121.14 (13)	C21—C22—C23	118.32 (14)
C3—C4—C4A	121.28 (14)	C21—C22—C25	121.34 (14)
C3—C4—H4	119.4	C23—C22—C25	120.32 (14)
C4A—C4—H4	119.4	C17—C18—H18A	109.5
C8—C7—C6A	110.09 (13)	C17—C18—H18B	109.5
C8—C7—H7A	109.6	H18A—C18—H18B	109.5
C6A—C7—H7A	109.6	C17—C18—H18C	109.5
C8—C7—H7B	109.6	H18A—C18—H18C	109.5
C6A—C7—H7B	109.6	H18B—C18—H18C	109.5
H7A—C7—H7B	108.2	C22—C25—H25A	109.5
C10—C9—C13	121.77 (15)	C22—C25—H25B	109.5
C10—C9—C8	122.42 (14)	H25A—C25—H25B	109.5
C13—C9—C8	115.81 (13)	C22—C25—H25C	109.5
C9—C10—C10A	122.90 (14)	H25A—C25—H25C	109.5
C9—C10—H10	118.6	H25B—C25—H25C	109.5
C10A—C10—H10	118.6	C14—C15—C16	112.86 (12)
C9—C8—C7	113.91 (13)	C14—C15—H15A	109.0
C9—C8—H8A	108.8	C16—C15—H15A	109.0
C7—C8—H8A	108.8	C14—C15—H15B	109.0
C9—C8—H8B	108.8	C16—C15—H15B	109.0
C7—C8—H8B	108.8	H15A—C15—H15B	107.8
H8A—C8—H8B	107.7	C18—C17—C16	112.87 (14)
O1—C6—C11	108.59 (12)	C18—C17—H17A	109.0
O1—C6—C12	103.19 (12)	C16—C17—H17A	109.0
C11—C6—C12	111.55 (13)	C18—C17—H17B	109.0
O1—C6—C6A	107.43 (11)	C16—C17—H17B	109.0
C11—C6—C6A	114.00 (12)	H17A—C17—H17B	107.8
C12—C6—C6A	111.40 (12)	C17—C16—C15	113.92 (13)
O1—C4A—C4	114.70 (13)	C17—C16—H16A	108.8
O1—C4A—C10B	123.32 (13)	C15—C16—H16A	108.8
C4—C4A—C10B	121.97 (13)	C17—C16—H16B	108.8
C10—C10A—C10B	115.39 (12)	C15—C16—H16B	108.8
C10—C10A—C6A	108.19 (11)	H16A—C16—H16B	107.7
C10B—C10A—C6A	109.85 (11)	C6—C12—H12A	109.5
C10—C10A—H10A	107.7	C6—C12—H12B	109.5
C10B—C10A—H10A	107.7	H12A—C12—H12B	109.5
C6A—C10A—H10A	107.7	C6—C12—H12C	109.5
C1—C10B—C4A	114.47 (13)	H12A—C12—H12C	109.5
C1—C10B—C10A	125.35 (13)	H12B—C12—H12C	109.5
C4A—C10B—C10A	120.18 (12)	C1—O2—S1	118.44 (8)
C24—C19—C20	121.03 (13)	C9—C13—H13A	109.5
C24—C19—S1	119.63 (11)	C9—C13—H13B	109.5
C20—C19—S1	119.31 (11)	H13A—C13—H13B	109.5
C21—C20—C19	118.91 (14)	C9—C13—H13C	109.5
C21—C20—H20	120.5	H13A—C13—H13C	109.5
C19—C20—H20	120.5	H13B—C13—H13C	109.5
C23—C24—C19	118.90 (13)	C6—C6A—C7	115.99 (12)
C23—C24—H24	120.5	C6—C6A—C10A	112.07 (12)
C19—C24—H24	120.5	C7—C6A—C10A	107.98 (12)
C20—C21—C22	121.40 (14)	C6—C6A—H6A	106.8
C20—C21—H21	119.3	C7—C6A—H6A	106.8
C22—C21—H21	119.3	C10A—C6A—H6A	106.8
C3—C14—C15	112.63 (12)	C4A—O1—C6	118.01 (11)
